# *Cronobacter sakazakii* infection alters serotonin transporter and improved fear memory retention in the rat

**DOI:** 10.3389/fphar.2015.00188

**Published:** 2015-09-04

**Authors:** Bhagavathi S. Sivamaruthi, Rajkumar Madhumita, Krishnaswamy Balamurugan, Koilmani E. Rajan

**Affiliations:** ^1^Department of Animal Science, School of Life Sciences, Bharathidasan University, Tiruchirappalli, India; ^2^Department of Biotechnology, Alagappa University, Karaikudi, India

**Keywords:** *Cron*o*bacter sakazakii*, animal model, fear memory, Hsp-90, SERT, microRNA-16

## Abstract

It is well established that *Cronobacter sakazakii* infection cause septicemia, necrotizing enterocolitis and meningitis. In the present study, we tested whether the *C. sakazakii* infection alter the learning and memory through serotonin transporter (SERT). To investigate the possible effect on SERT, on postnatal day-15 (PND-15), wistar rat pups were administered with single dose of *C. sakazakii* culture (infected group; 10^7^ CFU) or 100 μL of Luria-Bertani broth (medium control) or without any treatment (naïve control). All the individuals were subjected to passive avoidance test on PND-30 to test their fear memory. We show that single dose of *C. sakazakii* infection improved fear memory retention. Subsequently, we show that *C. sakazakii* infection induced the activation of toll-like receptor-3 and heat-shock proteins-90 (Hsp-90). On the other hand, level of serotonin (5-hydroxytryptamine) and SERT protein was down-regulated. Furthermore, we show that *C. sakazakii* infection up-regulate microRNA-16 (miR-16) expression. The observed results highlight that *C. sakazakii* infections was responsible for improved fear memory retention and may have reduced the level of SERT protein, which is possibly associated with the interaction of up-regulated Hsp-90 with SERT protein or miR-16 with SERT mRNA. Taken together, observed results suggest that *C. sakazakii* infection alter the fear memory possibly through SERT. Hence, this model may be effective to test the *C. sakazakii* infection induced changes in synaptic plasticity through SERT and effect of other pharmacological agents against pathogen induced memory disorder.

## Introduction

Serotonin (5-hydroxytryptamine, 5-HT) has been implicated as the modulator of learning and memory with special preference to consolidation of new information into long-term memory (Kandel, 2001). 5-HT play a key role in memory formation by interacting with other neurotransmitters/exerts its effect through their seven (5-HT_1_–5-HT_7_) subclass of receptors (Meneses, 1999, 2007, 2013; Meneses et al., 2011; Perez-Garcia and Meneses, 2009; Hoyer et al., 2002). Although evidence from *Aplysia* to human points at a functional role of serotonergic transmission in learning and memory, the underlying mechanism is depends on the level of 5-HT (Barbas et al., 2003; Meyer et al., 2009), and the depletion of 5-HT could affect the memory formation (Kaang et al., 1993; Barbas et al., 2003; Seyedabadi et al., 2014; Stansley and Yamamoto, 2015).

Serotonin transporters (SERT) play a key role in clearance of the released 5-HT through transport across pre-synaptic membrane and maintain the homeostasis of 5-HT level. In addition, expression status of SERT protein could control the duration and intensity of 5-HT activity at synapse (Gainetdinov and Caron, 2003; Tellez et al., 2012; Yoon et al., 2013; Bravo et al., 2014). Earlier studies reported that expression of SERT protein regulated by the interacting molecules such as ribonucleoprotein (RNP) and sequence specific microRNA (miR; Standart and Jackson, 1994; Wilkie et al., 2003; Bartel, 2009; Croce, 2009; Gyawali et al., 2010; Goldie and Cairns, 2012; Hartley et al., 2012). At this point, heterogeneous nuclear ribonucleoprotein K (hnRNPK) and miR-16 appears to negotiate for the binding site at 3′-untranslated region (UTR) of SERT and regulate the repression/depression of translation (Baudry et al., 2010; Yoon et al., 2013).

Over the past few years, research has been conducted to understand the pathogenicity mechanism, genetic, nature of survival and molecular characterization of virulence in *Cronobacter* spp. (Jaradat et al., 2014). Reports have shown that source of *Cronobacter* infection was the powder infant formula (PIF; Yan et al., 2012), apart from that significant association was found with contaminated home environment (Kandhai et al., 2004), packed foods (Friedemann, 2007) and drinking water (Liu et al., 2013). In parallel, Clinical and laboratory studies reported that they have resistance to heat, desiccation and acid stress growth condition (Breeuwer et al., 2003; Edelson-Mammel et al., 2005; Dancer et al., 2009), and *Cronobacter* infection in neonates and infants cause meningitis, necrotizing enterocolitis (NEC) and sepsis with case fatality rate ranging from 40 to 80% (Muytjens et al., 1983; Block et al., 2002; Hyun-Lee et al., 2011; Yan et al., 2012; Hunter and Bean, 2013). However, *Cronobacter* infection also have been reported in elder patients or immunocompromised persons (Healy et al., 2010), among them 50% had an underlying malignancy (Lai, 2001; See et al., 2007). In addition, *Cronobacter* infection have been linked to conjunctivitis, osteomyelitis, diarrhea, acute cholecystitis, and wound infection (Gosney et al., 2006; Flores et al., 2011; Yan et al., 2012; Tsai et al., 2013). Pathogen induced neuroinflammation can alter the behavior possibly either through hypothalamus pituitary-adrenal (HPA) axis or neurotransmitter system through the interacting molecules (Pérez et al., 2009; Herrero et al., 2015). In fact, several line of studies reporting that responding to the endotoxin (lipopolysaccharide, LPS) produced by the pathogenic bacteria, the host system activate innate immune response, in which different toll-like receptors (TLRs) and heat-shock proteins (Hsp) are part of it (Pandey and Agrawal, 2006; Okun et al., 2009; Chen et al., 2014), TLRs in dendritic cells play critical role (Stanislawska et al., 2004) and 5-HT transmission (Desbonnet et al., 2008; van Heesch et al., 2014; Depino, 2015). Currently, very little information is available on the pathogen infection mediated effect on serotonergic system. Therefore, the present study is designed to examine the effect of *Cronobacter sakazakii* infection on postnatal rats’ serotonergic system particularly on SERT and associated changes in learning and memory.

## Materials and Methods

### Bacterial Strain and Media

The bacterial strain *C. sakazakii* was obtained from American Type Cell Culture (ATCC BAA-894). The obtained bacterial strain was cultured on the selective chromogenic *Enterobacter sakazakii* agar medium (Song et al., 2008). The positive blue-green colonies were picked and grown on 1.5% Luria-Bertani (LB) agar. The overnight culture was prepared in the LB broth, which was maintained at 37°C in an incubator shaking at the rate of 145 rpm. Serial dilution and plating method was used to assess the bacterial concentration (Miller, 1972). In detail, 3 h culture of *C. sakazakii* was examined through biophotometer (Eppendorf Inc) at O.D_600·_ The bacterial culture was serially diluted and plated on LB agar for colony counting. Based on colony counting assay result, concentration of bacterial cells was calculated. Bacterial concentration of 10^7^ CFU was fixed as infectious dose for the present study based on LC_50_ analysis.

### Animals

Timed-pregnant wistar rats at gestation day-15 were acquired (Sri Venkateshwara Enterprise, Bangalore, India), acclimated and maintained under controlled ambiance (12 h light/dark cycle; temperature: 22 ± 2°C; humidity: 50 ± 5%). The pregnant rats were housed individually in a standard laboratory cage (43 cm × 27 cm × 15 cm) with saw dust as bedding material, and food and water provided *ad libitum*. This study was carried out in accordance with the recommendation of Institutional Animal Ethics Committee (IAEC), Bharathidasan University (BDU). The animal experimental protocol was approved by IAEC, BDU.

#### Experimental Groups

Wistar rat pups at the age of postnatal day-15 (PND-15) were used as host system for the present study. Pups from different litters were randomly divided into three different groups: naïve control (NC), medium control (MC), and infected (IF) group. Rat pups in NC groups were maintained at normal condition without treatment. MC groups were treated with single dose of LB (100 μL) and IF pups with *C. sakazakii* culture (10^7^ CFU) on PND-15 by oral gavage. Then the animals were maintained at typical condition with mother.

#### Confirmation of Infection

On PND-30, the amygdala region was dissected out as described by Kalin et al. (1994) from NC and IF group rats (*n* = 3 from each group) and homogenized in phosphate buffer saline (PBS). The homogenate was serially diluted up to 10^–4^ with PBS and plated on specific medium to identify *C. sakazakii* (Hicrome *E. sakazakii* agar; Himedia cat. No. M1641-100G) and incubated at 37°C for overnight to observe the presence of *C. sakazakii* in brain tissue.

#### Behavioral Test

***Passive avoidance test***

Passive avoidance apparatus was constructed following the specification of Zare et al. (2015). The apparatus consisted of equally sized light and dark compartments (20 cm × 40 cm × 20 cm) made up of Plexiglas separated by a guillotine door (12 cm × 12 cm). The floor of both chambers were made up of stainless steel rods (3 mm diameter) spaced 1 cm apart but the gridded floor of the dark chamber could be electrified using a shock generator. All the experiments were conducted between 09:00 and 18:00 h. All groups (NC, *n* = 14; MC, *n* = 20; IF, *n* = 29) were subjected to step-through passive avoidance test, in which the rats were trained to the criterion and tested for their retention 24 h post-training. Each time after removing the animal, the apparatus was wiped with 70% ethanol to remove odor. During each experiment the experimenter handle the animals for <60 s.

***Exploration and training***

On PND-31, each animal was placed in the light compartment of the apparatus facing away from the door and 10 s later the guillotine was raised. The animal was left for 5 min to habituate the apparatus. On PND-32, each animal was trained for the criterion. The rat was placed in the light compartment of the apparatus facing away from the door and 10 s later the guillotine was raised. When the animal had placed at all four paws in the dark compartment, entrance latency to the dark compartment was recorded. Once the animal entered into the dark compartment, the door was closed and an inescapable foot shock (0.5 mA) was applied for 5 s. After 20 s, the animal was retrieved from the dark box and placed back into their home cage. After 2 min, the procedure was repeated. The rat received foot shock each time it placed its four paws into the dark compartment. The training was terminated when the rat remained in light compartment for 120 s consecutively. Number of trials required for training the animal was recorded.

***Retention test***

On PND-33, retention test was performed 24 h post training. The rat was placed on the light compartment and 10 s later the door was raised. The step-through latency and time spent in dark compartment was recorded up to 300 s. If the rat did not enter the dark compartment within 300 s, a score of 300 s was assigned.

#### Neurotransmitter Analysis

On PND-30, group of rats from NC (*n* = 5), MC (*n* = 5), and IF (*n* = 5) were euthanized, and the amygdala region was dissected as described elsewhere (Kalin et al., 1994) and frozen on dry ice. The tissue samples were weighed and homogenized in a glass homogenizer with 0.1 M perchloric acid containing 4.5 mM Na_2_EDTA and 1.6 mM reduced glutathione. The homogenates were centrifuged at 12,000 rpm for 20 min at 4°C. The supernatants were collected in a fresh tube and stored at –70°C. The level of 5-HT was estimated with a 5-HT ELISA kit (Biosource, Europe S.A., Belgium) by following the manufacturer’s instructions. The concentrations of 5-HT in each tissue samples were calculated by comparing the optical density of the sample (mean for duplicates) with that of the standard curve.

#### Sample Preparation

On PND-30, group of rats from NC (*n* = 5), MC (*n* = 5), and IF (*n* = 5) groups were euthanized and amygdala region was dissected out from and divided into two part for the preparation of total RNA and protein. Total RNA was isolated from the tissue samples following the manufacture’ instructions (Trizol method; Merck, Bangalore, India) and stored at –70°C with RNase inhibitor (1U/μL; Rnasin, Promega, Madison, WI, USA). Total RNA (1 μg) was converted into cDNA by following manufacture’ instructions (QuantiTect^®^ Reverse Transcription Kit; catalog no. 205311, Qiagen, Germany). Tissue samples were homogenized in 300–400 μL of ice cold lysis buffer (150 mM NaCl, 50 mM Tris–HCl; pH 7.5, 5 mM EDTA, 0.1% v/v NP-40, 1 mM DTT, 0.2 mM sodium orthovanadate, 0.023 mM PMSF) with protease inhibitor cocktail (10 mg/mL; Sigma-Aldrich, USA), and incubated on ice for 30 min. The homogenate was centrifuged at 10,000 g for 30 min at 4°C. The supernatant was collected in a fresh tube and again centrifuged at 12,000 g for 30 min at 4°C. The supernatant was extracted and stored at –70°C.

#### Quantitative Real-Time PCR

The quantitative real-time PCR (qRT-PCR) was performed in CFX-96 Touch™ Real-time PCR detection system using SSoAdvanced™ SYBR^®^ green mix (Bio-Rad Laboratories, Inc., USA). The level of mRNA of the selected genes were assessed through qPCR using specific primers: *Tlr-3* (for 5′-ACAATGCCCAACTGAACCTC-3′ and rev 5′-CGGAGGCTGTTGTAGGAAAG-3′) and *miR-16* (for 5′-CCGCTCTAGCAGCACGTAAA-3′ and rev 5′-CCCTGTCACACT AAAGCAGC-3′). The level of *Tlr-3* and *miR-16* was normalized with internal control GAPDH (for 5′-AACATCATCCCTGCATCCAC-3′ and rev 5′-AGGAACACGGAAGGCCAT GC-3′) and U6 SnRNA (for 5′-CTCGCTTCGGCAGCACA-3′ and rev 5′-AACGCTTCACGAATT TGCGT-3′), respectively. Thermo cycling conditions for qPCR were as follows: initial denaturation at 92°C for 3 min and then denaturation at 92°C for 5 s, annealing (at 59°C for GAPDH, 62°C for *tlr-3*, 60°C for *U6 SnRNA*, and 64°C for *miR-16*), for 5 s, extension at 72°C for 5 s, and melt curve analysis at 65–95°C. Amplification of the single PCR product was confirmed by monitoring the dissociation curve followed by melting curve analysis. Each reaction was performed in triplicates with threefold serial dilution of cDNA with normalizing internal control GAPDH/U6 SnRNA. The data are presented as mean fold change of the normalized expression (CFX Manager™ version 2 software; Bio-Rad Laboratories, Inc., USA).

#### Western Blotting

An equal concentration of protein (40 μg) was mixed with loading buffer (glycerol, 125 mM Tris–HCl pH 6.8, 4% SDS, 0.006% bromophenol blue, 2% mercaptoethanol) and resolved on 10% polyacrylamide gel (PAGE). The separated proteins were transferred electrophoretically on to the PVDF membrane (Millipore India Pvt. Ltd., India). The membranes were then placed in the blocking solution [5% non-fat dry milk in Tris-buffered saline (TBS) containing 0.1% Tween-20: TBS-T] for 3 h at room temperature (RT). The blocking solution was discarded and the membranes incubated at 4°C overnight with one of the following primary antibodies (Santa-Cruz Biotech, Germany/BD Biosciences, USA): SERT (SC-1458, 1:200), anti-β-actin (SC-130656; 1:1000) affinity purified rabbit polyclonal antibody and Hsp-90 (SC-5977, 1:200) mouse monoclonal antibody. β-actin was used as control for each samples. The membrane was washed and bound antibodies were detected by incubating for 3 h either with the mouse anti-rabbit (Cat # 621100180011730; 1:2000; MERCK, Bangalore, India) or goat anti-mouse (Cat # 621100480011730; 1:2000; MERCK, Bangalore, India) alkaline phosphatase conjugated antibody. The membrane was washed three times with TBS-T, and alkaline phosphatase activity was detected with 5-bromo-4-chloro-3-indolylphosphate disodium salt (BCIP)/nitro-blue tetrazolium chloride (NBT) following the instructions from the manufacturer (Invitrogen, USA). The images were acquired with Molecular Imager ChemiDoc XRS system (Bio-Rad Laboratories, Inc., USA) and the trace quantity for each band was measured using Quantity One image analysis software (Bio-Rad Laboratories, Inc., USA). The obtained Hsp90 and SERT levels were normalized to β-actin for respective samples.

#### Statistical Analysis

Data were presented as a mean ± standard error of the mean (SEM) and plotted with KyPlot (version 1.0) for graphical representation. The obtained data were evaluated by one-way analysis of variance (ANOVA) to detect differences between groups (SigmaStat; version 3.1) followed by Bonferroni *post hoc* test was performed. Differences were considered significant if *p* < 0.05.

## Results

### *C. sakazakii* Infection Alters the Fear Memory Retention

**FIGURE 1:**
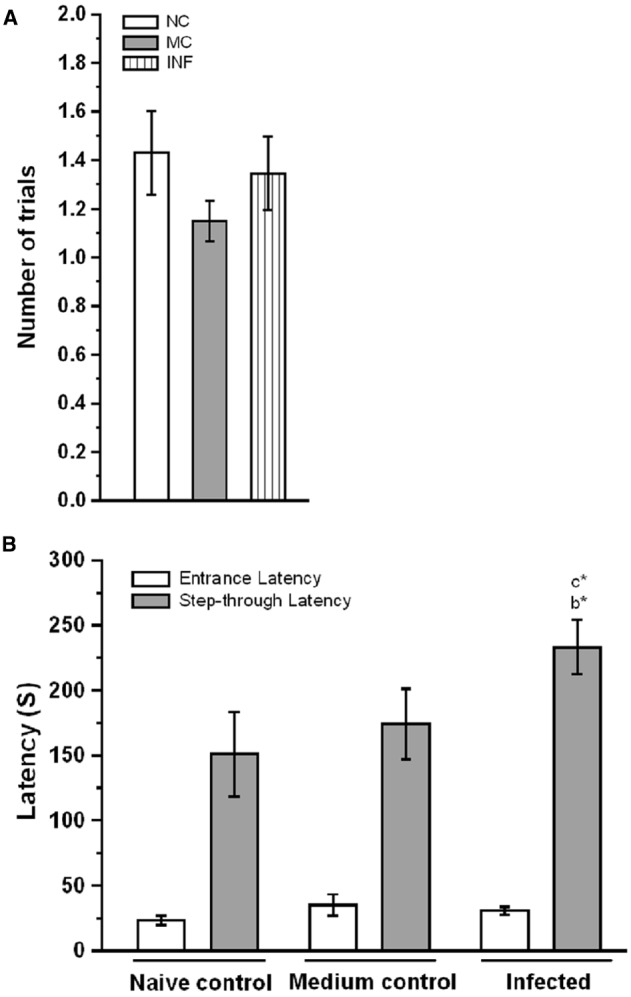
*****Cronobacter sakazakii*** infection improved the fear memory retention in passive avoidance (PA) test. (A)** The number of trials to acquisition, naïve control (NC), medium control (MC), and infected (IF) group did not show significant difference in the number of trials to acquisition. **(B)** Step-through latency in the retention test. IF group rats spend more in the light-chamber and taken more time to step-down into the dark-chamber considered as indices of improvement in fear memory. Values are represented as the mean ± SEM; *P < 0.05. Asterisk indicates significant difference respect to comparison between groups (b = NC verses IF; c = MC verses IF).

To determine whether the *C. sakazakii* infection affects cognitive function, we compare the fear memory retention between the experimental groups. We first assessed the performance of experimental groups during the training session of the light-dark passive avoidance task, there was no significant difference in the number of acquisition trials between NC (1.42 ± 0.17) and MC (1.15 ± 0.08) groups [*F*_(1,33)_ = 2.56; *P* > 0.05]. Similarly, *C. sakazakii* infection did not change the number of trials in IF group (1.35 ± 0.15) from NC [*F*_(1,42)_ = 0.113; *P* > 0.05] and MC group [*F*_(1,47)_ = 1.00; *P* > 0.05; Figure 1A]. Further, Bonferroni test revealed that the acquisition trials required by IF group was not significantly different from NC (*P* = 0.739) and MC group (*P* = 0.322). In comparison, there was no significant difference between NC and MC groups (*P* = 0.011). Similarly, there was no significant difference in entrance latency between NC (23.21 ± 3.36 s) and MC (35.10 ± 8.13 s) group [*F*_(1,32)_ = 1.36; *P* > 0.05]. When the IF group (30.62 ± 3.0 s) compared to NC [*F*_(1,42)_ = 2.26; *P* > 0.05] and MC [*F*_(1,47)_ = 0.344; *P* > 0.05], no significant difference was found (Figure 1B). Bonferroni test confirmed that the entrance latency of IF group was not significantly different from NC (*P* = 0.144) and MC group (*P* = 0.62). In addition, it showed that NC group was not significantly different from MC group (*P* = 0.160). However, there was a significant difference between groups in step-through latency during testing. Our analysis revealed that the IF group (237.34 ± 19.35 s) rats showed significantly higher latencies to enter the dark box compared to NC (150.92 ± 30.5 s) [*F*_(1,42)_ = 4.883; *P* < 0.05] and MC (171.8 ± 26.38 s) group [*F*_(1,47)_ = 4.26; *P* < 0.05]. When we compare the latency exhibited by the NC and MC groups, there was no significant difference between them [*F*_(1,32)_ = 0.344; *P* > 0.05]. Further, Bonferroni test showed that the IF group took significantly more time to step into the dark box than NC (*P* = 0.021) and MC group (*P* = 0.044), but there was no significant difference between NC and MC group (*P* = 0.596). The observed data showed that *C. sakazakii* infection did not alter their learning during acquisition but IF group exhibited higher step-through latency during retention test, which showed the persistence of fear memory.

#### *C. sakazakii* Entered into the Brain

**FIGURE 2:**
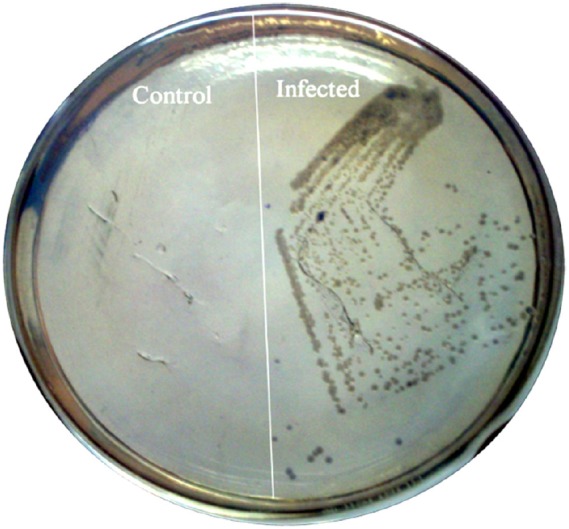
*****Cronobacter sakazakii*** colonization in the brain tissue of rat pups.** Brain tissues were obtained from experimental groups (Control, Infected) on PND-30. Equal weights of brain tissues were homogenized and homogenates were plated on specific medium. Specific medium showing the presence of *C. sakazakii* in infected group.

To confirm the observed behavioral phenotype was due to the single dose of *C. sakazakii* infection on PND-15, we tested the presence of *C. sakazakii* in brain. When we plated the brain tissue homogenates in *Enterobacter* medium plate, we found the growth of blue-green color colonies from the IF group samples but not in the naïve control (Figure 2). This result suggested that single dose of oral treatment of *C. sakazakii* during post-natal day is enough to induce the infection at brain.

#### *C. sakazakii* Infection Activates TLR-3

**FIGURE 3:**
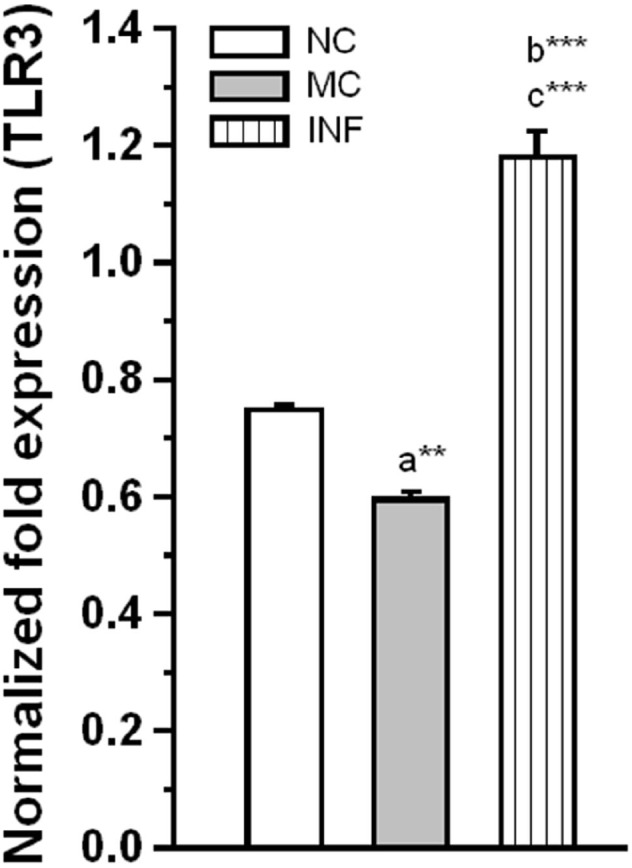
**Rats received ***C. sakazakii*** infection showed up-regulated expression of TLR-3.** The level of TLR-3 expression was significantly increased in infected (IF) group than medium control (MC) and naïve control (NC). The normalized fold variation shown as mean ± SEM. Asterisk indicates significant difference (**P < 0.01; ***P < 0.001) respect to comparison between groups (a = NC verses MC; b = NC verses IF; c = MC verses IF).

To further evaluate the effect of *C. sakazakii* infection on activation of TLR-3. Our analysis revealed that *C. sakazakii* infection significantly increased the expression level of TLR-3 (Figure 3), the Ct values of TLR-3 for each group followed by GAPDH (NC: 23.24 ± 0.038; 12.11 ± 0.041; MC: 23.24 ± 0.026; 11.77 ± 0.064; IF: 22.36 ± 0.122; 12.12 ± 0.054). The estimated level of TLR-3 was significantly higher in IF group than MC group [*F*_(1,9)_ = 167.19; *P* < 0.001] and NC group [*F*_(1,9)_ = 103.78; *P* < 0.001]. Similarly, there was a significant difference between MC and NC groups, but the difference obtained by the reduction of TLR-3 expression was significant in MC group than NC group [*F*_(1,9)_ = 23.68; *P* < 0.01]. These results suggesting that *C. sakazakii* infection activated TLR-3 expression.

#### *C. sakazakii* Infection Up-Regulate Hsp-90

**FIGURE 4:**
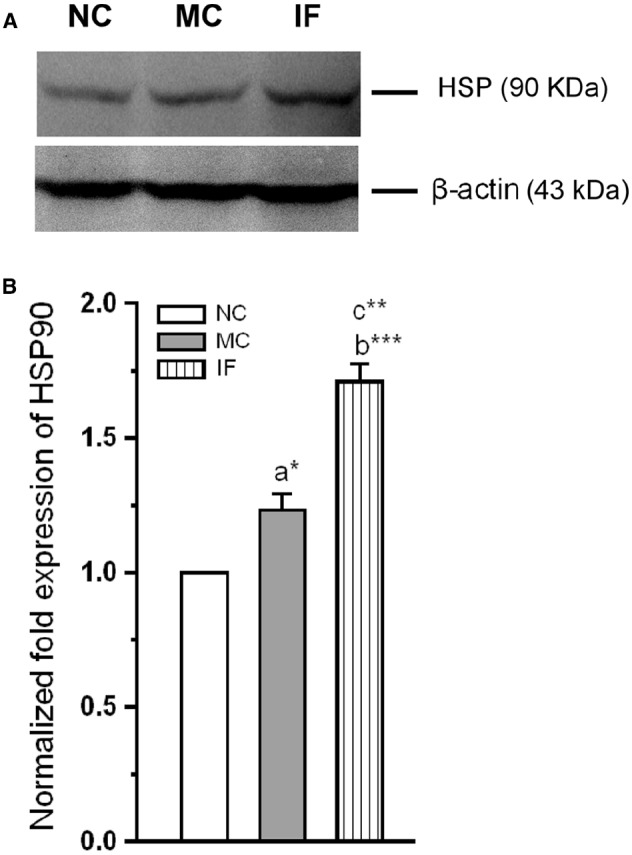
*****Cronobacter sakazakii*** infection increases the expression of heat-shock protein-90. (A)** Representative western blot showing variation in the expression of HSP-90 levels in experimental groups. **(B)** HSP-90 level was significantly increased in infected (IF) group than control (MC) and naïve control (NC) groups. The fold variation shown as mean ± SEM. Asterisk indicates significant difference (*P < 0.05; **P < 0.01; ***P < 0.001) respect to comparison between groups (a = NC verses MC; b = NC verses IF; c = MC verses IF).

We next examined along with activation TLR-3, whether the Hsp-90 also activated following *C. sakazakii* infection. When examined the level of Hsp-90 in the experimental groups (Figure 4), we found that the *C. sakazakii* infection significantly alter the Hsp-90. The estimated level was significantly high in IF group than MC [*F*_(1,9)_ = 188.64; *P* < 0.001] and NC group [*F*_(1,9)_ = 1424.96; *P* < 0.001]. However, there was no significant difference between MC and NC groups [*F*_(1,9)_ = 6.28; *P* = 0.052]. Our analysis revealed that *C. sakazakii* increased Hsp-90 expression.

#### *C. sakazakii* Infection Modulates Serotonin and SERT Protein Level

**FIGURE 5:**
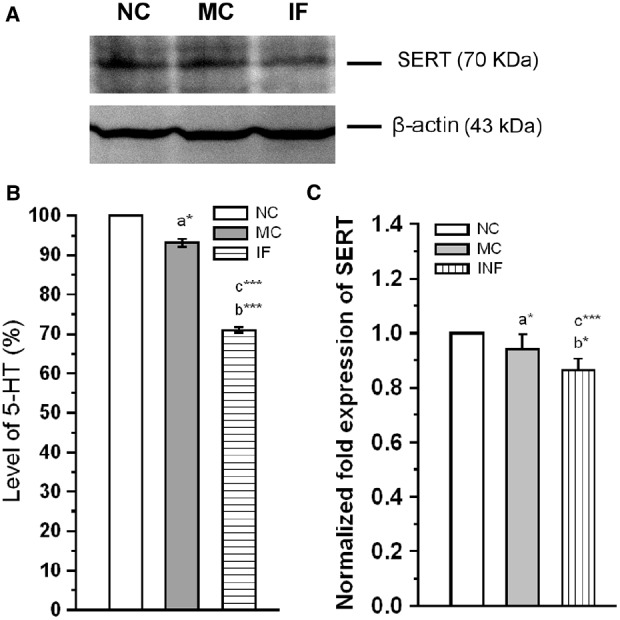
*****Cronobacter sakazakii*** infection alters the serotonergic system. (A)** Representative western blot showing the differential expression of SERT in experimental groups. **(B)** There was a significant decrease in 5-HT level in *C. sakazakii* infected (IF) group than medium control (MC) and naïve control (NC) groups. (C) SERT protein level was significantly decreased in IF group than MC and NC mice. Data were shown as mean ± SEM. Asterisk indicates significant difference (*P < 0.05; ***P < 0.001) respect to comparison between groups (a = NC verses MC; b = NC verses IF; c = MC verses IF).

In addition to the activation of TLR-3 and Hsp-90, we estimated the level of 5-HT, and expression level of SERT in experimental group rats. As shown in Figure 5, the basal levels of 5-HT was significantly affected by *C. sakazakii* infection [*F*_(1,9)_ = 9735.27; *P* < 0.001] compared to NC and MC [*F*_(1,9)_ = 236.78; *P* < 0.001]. In addition, levels of 5-HT was significantly lower in MC group than NC group [*F*_(1,9)_ = 9.12; *P* < 0.05]. Further, our analysis revealed that the expression of SERT was significantly reduced in IF group [*F*_(1,9)_ = 51.85; *P* < 0.001] than NC group, but not significantly different from MC group [*F*_(1,9)_ = 4.8; *P* = 0.07]. When we compare the expression level of MC and NC groups, they were not significantly different [*F*_(1,9)_ = 4.1; *P* = 0.074]. Our analysis suggests that *C. sakazakii* infection reduced the levels of 5-HT and expression of SERT protein.

#### *C. sakazakii* Infection Modulates miR-16 Expression

**FIGURE 6:**
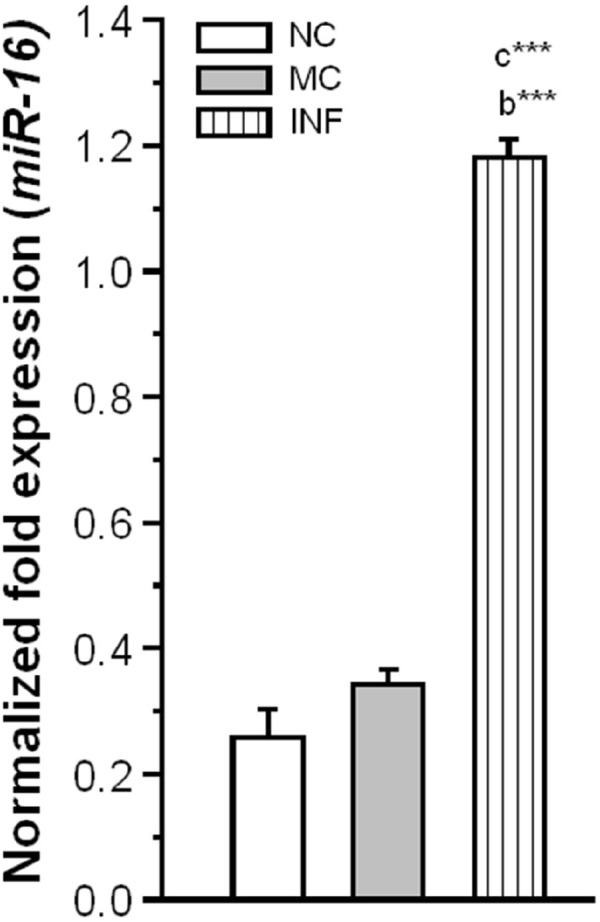
*****Cronobacter sakazakii*** infection alters the miR-16 expression.** The level of miR-16 expression was significantly increased in infected (IF) group than control (MC) and naïve control (NC) groups. The normalized fold variation shown as mean ± SEM. Asterisk indicates significant difference (***P < 0.001) respect to comparison between groups (a = NC verses MC; b = NC verses IF; c = MC verses IF).

Subsequently, we explored the possible role of miR-16 in *C. sakazakii* mediated regulation of SERT protein expression. We observed that the level of miR-16 expression was significantly elevated in IF (Ct: 23.21 ± 0.054) group than NC [*F*_(1,9)_ = 8348.15; *P* < 0.001] and MC group [*F*_(1,9)_ = 8117.98; *P* < 0.001]. However, there was no significant difference between the MC (Ct: 24.73 ± 0.103) and NC (Ct: 25.34 ± 0.260) groups [*F*_(1,9)_ = 1.68; *P* = 0.084; Figure 6]. Our results suggested that up-regulation of miR-16 by *C. sakazakii* infection possibly suppressed the translation of SERT.

## Discussion

*Cronobacter sakazakii* has been associated with human infection especially in newborn and infants (Joseph and Forsythe, 2011; Cruz-Córdova et al., 2012). Earlier, Hyun-Lee et al. (2011) demonstrated that *C. sakazakii* can cross the blood–brain barrier (BBB) in postnatal mice (PND-3.5), possibly through exploiting immature dendritic cells (Townsend et al., 2007; Mittal et al., 2009; Emami et al., 2011). In the present study, we IF the postnatal rats on PND-15, during the onset of “brain growth spurt” period (Dobbing and Sands, 1979). During this period, changes like axonal outgrowth and dendritic maturation, establishment of neuronal connections and proliferation of glial cells occurred accompanying with myelinization (Kolb and Whishaw, 1989). At first, we showed the presence of *C. sakazakii* in PND-30 rats’ brain. Further, we demonstrated that single dose of *C. sakazakii* infection in postnatal rats did not alter their learning efficiency but improved the fear memory retention. Our results adding support to the earlier study and suggest that postnatal Wistar rats may be used as animal model for human neonatal *C. sakazakii* infections. Earlier studies discussed how the infection and inflammation lead to changes in brain (Goehler et al., 2007; Jaradat et al., 2014), in which TLRs are a part. TLRs are conserved from sponges to human and very much present in neuronal cells (Barton, 2007; Tang et al., 2007; Wiens et al., 2007). It has been stated that as a pro-inflammatory or a comprehensive neuroprotective response, TLR-3 is activated (Bsibsi et al., 2006; Kim et al., 2008), whereas its activation has not yet been established under normal condition (Okun et al., 2011). In brain, TLR-3 has broad effect on the cognitive function based on injury and/or disease. Studies in animal models reported that TLR-3 deficient mice showed improved contextual and extinction of fear memory (Okun et al., 2010). Interestingly, we found that rats with *C. sakazakii* infection showed elevated level of TLR-3 expression compared to other groups and they displayed improved fear memory. Supporting to this, earlier study demonstrated that TLR-3 activation possibly negatively regulate ERK-CREB signaling, thus, activation of TLR-3 contribute to cognitive impairment and other behavioral disorders (Okun et al., 2010).

Earlier studies reported that as a innate immune response exposure to pathogen/LPS activate TLR and Hsp90 (Stanislawska et al., 2004; Xie et al., 2015), in many observations expression of Hsp90 facilitates the pathogenesis (Qin et al., 2010; Smith et al., 2010; Shapiro et al., 2012). TLR-3 can also respond to the endogenous ligands such as Hsp-90, especially in dendritic cells during pathogenesis (Stanislawska et al., 2004). Similarly, we found that *C. sakazakii* infection induced the expression of Hsp90, the estimated level was higher than the other experimental group. Hsp90 is one of the molecules that interact with serotonergic system, especially with SERT. In fact, N- or C-terminus of SERT protein known to interact with many regulatory proteins, they play critical role in folding of SERT protein (El-Kasaby et al., 2010, 2014; Zhong et al., 2012). When we tested the expression pattern of SERT, the level of SERT protein was significantly low in IF group than other experimental groups. Although, it is interesting that the infection appears to have changed 5-HT level and SERT protein, the SERT effect appeared to be rather modest and it is unclear whether the alternation of SERT or any other interacting molecules in altering the individual’s behavior in IF group. However, earlier *in vitro* report demonstrated that over expression of Hsp90 interact with SERT protein and alter the folding trajectory of SERT protein (El-Kasaby et al., 2014). On the other hand, expression of SERT could be exerted by microRNAs, particularly miR-16 (Baudry et al., 2010; Yoon et al., 2013). Specific miRNAs activation/inactivation patterns are critically regulated by the presence of bacterial effector proteins and localization of the pathogen (Zhu et al., 2010; Al-Quraishy et al., 2012; Izar et al., 2012). Although, there is a differential expression of miR-16 following pathogen infection, we found that miR-16 expression was increased after *C. sakazakii* infection. Further, our analysis suggests that *C. sakazakii* infection up-regulate the expression of miR-16, which also interact with the 3′UTR of SERT and down-regulate the translation process. Supporting to our behavioral observations, SERT knock-out animals showed impaired fear extinction (Wellman et al., 2007; Narayanan et al., 2011; Hartley et al., 2012). The down-regulated SERT expression could affect the reuptake of released 5-HT, and then the level of 5-HT. Our analysis revealed that the level of 5-HT significantly decreased following *C. sakazakii* infection. Supporting to this, *in vivo* and *in vitro* studies demonstrating that exposure to pathogens/pathogen produced endotoxin alter the level of 5-HT and behavior (Esmaili et al., 2009; Martin et al., 2009; Shin and Liberzon, 2010; van Heesch et al., 2014). In addition, we observed difference between the naïve control and MC in molecules we tested in this study but not in the behavior. The observed difference in this study is possibly by the micronutrients in the bacterial medium, which may alter the gut microbiota of the individuals. They have the capacity to can influence precursor pool for 5-HT (Desbonnet et al., 2008).

In conclusion, our results demonstrates that *C. sakazakii* infection enhanced the fear memory retention. Although, further study needed to establish the mechanism of this effect, based on our data, we hypothesize that observed changes in SERT expression may have caused this effect, possibly through the interaction of Hsp-90 and miR-16. Further, the present study suggest that *C. sakazakii* infection in postnatal rats may be used an animal model to examine the effect of bacterial infection mediated changes in synaptic plasticity through SERT and effect of other pharmacological agents against pathogen induced memory disorder.

### Conflict of Interest Statement

The authors declare that the research was conducted in the absence of any commercial or financial relationships that could be construed as a potential conflict of interest.
